# Numerical Study on the Buckling Behavior of FG Porous Spherical Caps Reinforced by Graphene Platelets

**DOI:** 10.3390/nano13071205

**Published:** 2023-03-28

**Authors:** Zhimin Zhou, Yun Wang, Suying Zhang, Rossana Dimitri, Francesco Tornabene, Kamran Asemi

**Affiliations:** 1 Hangzhou Vocational & Technical College, Hangzhou 310018, China; 2 Department of Innovation Engineering, Faculty of Engineering, University of Salento, 73100 Lecce, Italy; 3 Department of Mechanical Engineering, Islamic Azad University, North Tehran Branch, Tehran 1477893855, Iran

**Keywords:** buckling, FEM, functionally graded materials, graphene platelets, porous materials, spherical caps, 3D elasticity

## Abstract

The buckling response of functionally graded (FG) porous spherical caps reinforced by graphene platelets (GPLs) is assessed here, including both symmetric and uniform porosity patterns in the metal matrix, together with five different GPL distributions. The Halpin–Tsai model is here applied, together with an extended rule of mixture to determine the elastic properties and mass density of the selected shells, respectively. The equilibrium equations of the pre-buckling state are here determined according to a linear three-dimensional (3D) elasticity basics and principle of virtual work, whose solution is determined from classical finite elements. The buckling load is, thus, obtained based on the nonlinear Green strain field and generalized geometric stiffness concept. A large parametric investigation studies the sensitivity of the natural frequencies of FG porous spherical caps reinforced by GPLs to different parameters, namely, the porosity coefficients and distributions, together with different polar angles and stiffness coefficients of the elastic foundation, but also different GPL patterns and weight fractions of graphene nanofillers. Results denote that the maximum and minimum buckling loads are reached for GPL-X and GPL-O distributions, respectively. Additionally, the difference between the maximum and minimum critical buckling loads for different porosity distributions is approximately equal to 90%, which belong to symmetric distributions. It is also found that a high weight fraction of GPLs and a high porosity coefficient yield the highest and lowest effects of the structure on the buckling loads of the structure for an amount of 100% and 12.5%, respectively.

## 1. Introduction

Nowadays, there is a high demand for materials with a low weight and high strength, for many engineering applications. Among them, FG-GPL porous materials have attracted the interest of many researchers due to their mechanical potentials in aerospace and marine industries. A large variety of works from the scientific literature have focused on the static and/or dynamic behavior of different structural members, such as beams, plates, shells, with arbitrary shapes and made of composite materials [1,2,3,4]. For example, Zhang et al. [5] applied the DSC-regularized Dirac-delta method using the Timoshenko theory to explore the dynamics of FG-GPL porous beams resting on elastic foundations and subjected to a moving load. Based on a shear and normal deformation theory and by employing the Ritz approach, Priyanka et al. [6] investigated the stability and dynamic responses of porous beams made of FG-GPLs. Moreover, the free vibrations of rotating, FG-GPL, porous Timoshenko beams were studied by Binh et al. [7], using the generalized differential quadrature method (GDQM). Xu et al. [8] adopted the differential transformation method to investigate the free vibration behavior of FG-GPL porous beams based on the Euler–Bernoulli beam theory under a spinning movement. Ganapathi et al. [9] proposed a trigonometric shear deformation theory, including a thickness stretching effect, to study the dynamic problem of curved beams made of FG-GPL porous nanocomposites, and proposed a closed-form solution as valid tool for further computational investigations. Yas and Rahimi [10] applied the GDQM to study the thermal vibration of FG-GPL, porous Timoshenko beams. Safarpour et al. [11] applied the 3D elasticity theory in conjunction with the GDQM to study the bending and free vibration behavior of porous annular and circular plates made of FG-GPLs under different boundary conditions. A novel computational method was proposed by Nguyen et al. [12] to evaluate the static bending and free vibration response of FG-GPL porous plates based on a first-order shear deformation theory (FSDT), while using a polygonal mesh with parabolic shape functions. Furthermore, the nonlinear free vibrations of porous plates made of FG-GPL nanocomposites, resting on an elastic foundation, were investigated using the GDQ approach by Gao et al. [13], using classical plate theory (CPT) and von Kármán-type nonlinearities. The same FSDT basics were applied by Saidi et al. [14] to study analytically the stability and vibrations of FG-GPL porous plates under an aerodynamical loading. The classical finite element approach and Rayleigh-Ritz procedure for a comprehensive investigation of the free and forced vibration behavior, and the static response of FG-GPL porous annular sector plates, were considered by Asemi et al. [15] using an FSDT approach. In addition, Phan [16] applied a refined plate theory to analyze the free and forced vibrations of porous plates made of FG-GPL nanocomposites, while using the (NURBS) non-uniform rational B-spline approximations. An analytical solution to the wave-propagation problem of FG-GPL porous plates was presented by Gao et al. [17], based on different plate theories, such as CPT, FSDT, or higher order theories (HSDTs). Zhou et al. [18] combined the 3D elasticity theory and GDQM to assess the free vibrations of FG-GPL porous plates, whereas in Ref. [19], the authors proposed a multiple scale approach and Galerkin method in order to define the nonlinear, forced vibration response of porous, thin, rectangular plates made of FG-GPL nanocomposites, including the von Kármán-type nonlinearities. Furthermore, a deep review on FG-GPL porous structures was performed by Kiarasi et al. [20]. The fabrication issues of these structures represent a challenging aspect for many practical applications. A novel quadrilateral element was proposed by Ton-That et al. [21], in line with the FSDT and Chebyshev polynomials, to analyze FG-GPL porous plates/shells. In addition, a variational differential quadrature (VDQ) was proposed by Ansari et al. [22] for solving the free-vibration response of post-buckled, arbitrarily shaped porous plates made of FG-GPL nanocomposites, based upon a third-order shear deformation theory (TSDT). The static and free-vibration analysis of FG-GPL annular plates, cylindrical shells and truncated conical shells, with various boundary conditions, within a three-dimensional elasticity theory, were also investigated by Safarpour et al. [23]. Bahaadini [24] defined a further analytical solution to the free vibration problem of FG-GPL, porous, truncated conical shells, according to a Love’s first approximation theory, while examining the influences of porosity coefficients, weight fractions and geometries of GPLs, on the free vibration of the structure. Babaei and his coauthors analyzed the stress-wave propagation and natural frequencies of porous joined conical-cylindrical shells made of FG-GPLs [25] and joined conical-cylindrical-conical shells [26] by using the classical finite element method (FEM). Based on the Donnell’s theory and the Galerkin approach, the internal resonance of metal foam cylindrical shells made of FG- GPLs was studied by Ye and Wang [27]. In the further work [28], the authors employed the Galerkin method and an improved version of Donnell nonlinear shell theory to investigate the nonlinear vibration of metal foam cylinders reinforced with GPLs. Moradi et al. [29] applied the moving least squares (MLSs) interpolations using an axisymmetric model to analyze stress waves’ propagation in FG-GPL, porous, thick cylinders in a thermal gradient environment. Based on the FSDT, Salehi et al. [30] solved analytically the nonlinear vibration of imperfect, FG-GPL, porous nanocomposite cylindrical shells, whereas in Ref. [31] the authors applied the GDQM to investigate the free vibration of sandwich pipes, considering the effects of porosity and a GPL reinforcement on the conveying fluid flow. Among the recent literature, Zhou et al. [32] examined the flutter and vibration properties of FG-GPL, porous cylindrical panels under a supersonic flow. At the same time, the vibration of FG-GPL porous shells was analytically investigated by Ebrahimi et al. [33]. Pourjabari et al. [34] analytically investigated the effect of porosity on the free and forced-vibration characteristics of GPL-reinforcement composite cylindrical shells in a nonlocal sense, based on a modified strain gradient theory (MSGT). In line with the previous works, a limited attention has been paid to the buckling response of FG-GPL porous materials and structures. Among the available literature, Zhou et al. [35] presented an accurate nonlinear buckling study of FG-GPL, porous, composite cylindrical shells based on Donnell’s theory and HSDT. Shahgholian-Ghahfarokhi et al. [36,37] investigated the torsional buckling behavior of FG-GPL, porous cylindrical shells, according to a FSDT and Rayleigh-Ritz method. Similarly, Yang [38] applied the Chebyshev polynomials-based Ritz method to study the natural frequencies and buckling response of FG-GPL porous rectangular plates, using the FSDT approach. Dong [39] investigated the buckling behavior of spinning cylindrical shells made of FG-GPL porous nanocomposites, while applying a FSDT and Galerkin approach. A novel numerical DQ-FEM solution to investigating the buckling and post-buckling of FG-GPL porous plates with different shapes and boundary conditions was applied by Ansari et al. [40]. Kitipornchai [41] analyzed the natural frequencies and elastic buckling of FG-GPL porous beams using the Timoshenko beam approach and the Ritz method. Twinkle et al. [42] focused on the impacts of grading, porosity and edge loads on the natural frequency and buckling problems of porous cylindrical panels made of FG-GPLs. Nguyen [43] investigated the buckling, instability and natural-frequency response of FG porous plates reinforced by GPLs using three-variable higher order isogeometric analysis (IGA). Rafiei Anamagh and Bediz [44], instead, applied the FSDT to study the free vibration and buckling behavior of porous plates made of FG-GPLs using a spectral Chebyshev approach.

In the available literature, it seems that the static, buckling and dynamic behavior of porous spherical shells made of FG-GPLs has not been surveyed so far, despite their geometry being of great interest in various engineering applications, such as heat exchangers or energy absorbers, among other applications in the areas of aerospace, mechanical engineering and marine engineering. Among the different shell geometries, a spherical shell structures, indeed, features a high strength with a simple geometry, even compared to a cylindrical structure. The design of such structural members considering only static loading conditions may fail in dynamic situations. In such context, we focus on the buckling capacities of spherical shells made of porous FG nanocomposites reinforced by graphene, due to their exceptional flexibility and enhanced physical features. It is well known from the literature, indeed, that porous ceramic nanocomposites can ensure different beneficial effects, such as a reduced electrical and thermal conductivity; low weight; reasonable hardness; and resistance to wear, corrosion and high-temperature applications [45]. Among the few works on spherical shell dynamics available in the literature, we cite Refs. [46,47], where a Ritz-Galerking procedure was proposed to solve a dynamic buckling problem for clumped spherical members. A finite difference method was applied, instead, in [48,49,50], to check for the sensitivity of the dynamic buckling response of spherical caps to some initial manufacturing imperfections. Novel theoretical shear deformation theories were applied in Refs. [51,52] to treat the buckling response of isotropic and orthotropic shallow spherical caps, whose problem was solved analytically by means of Chebychev series [51], or numerically according to classical finite elements [52]. At the present state, however, there is a general lack of works from the literature focusing on the dynamic buckling of GPL-reinforced porous nanocomposite spherical shells, whose aspects are explored here according to the 3D elasticity basics and Green deformation nonlinearities, rather than common shell theories and Von-Karman nonlinearities, as proposed in [53].

The equilibrium equations of a pre-buckling state are determined from the principle of virtual work, whose solution is found according to classical finite elements. The buckling loads are obtained according to the nonlinear Green strain field and the generalized geometric stiffness concept, for spherical caps featuring a uniform and non-uniform pattern of GPLs in the metallic matrix, including open-cell internal pores and for various porosity distributions along the shell’s thickness with uniform and symmetric FG patterns. More specifically, five different patterns of GPL dispersion pattern are assumed throughout the shell’s thickness, namely, a FG GPL-X, A, V, UD and O patterns. A systematic investigation checks for the effects of various porosity distributions and GPL patterns, along with the weight fractions and porosity coefficients of nano-fillers and different polar angles, on the buckling behavior of FG-GPL, porous spherical caps.

## 2. Theoretical Problem

### 2.1. Description of Geometry and Mechanical Properties

**Figure 1:**
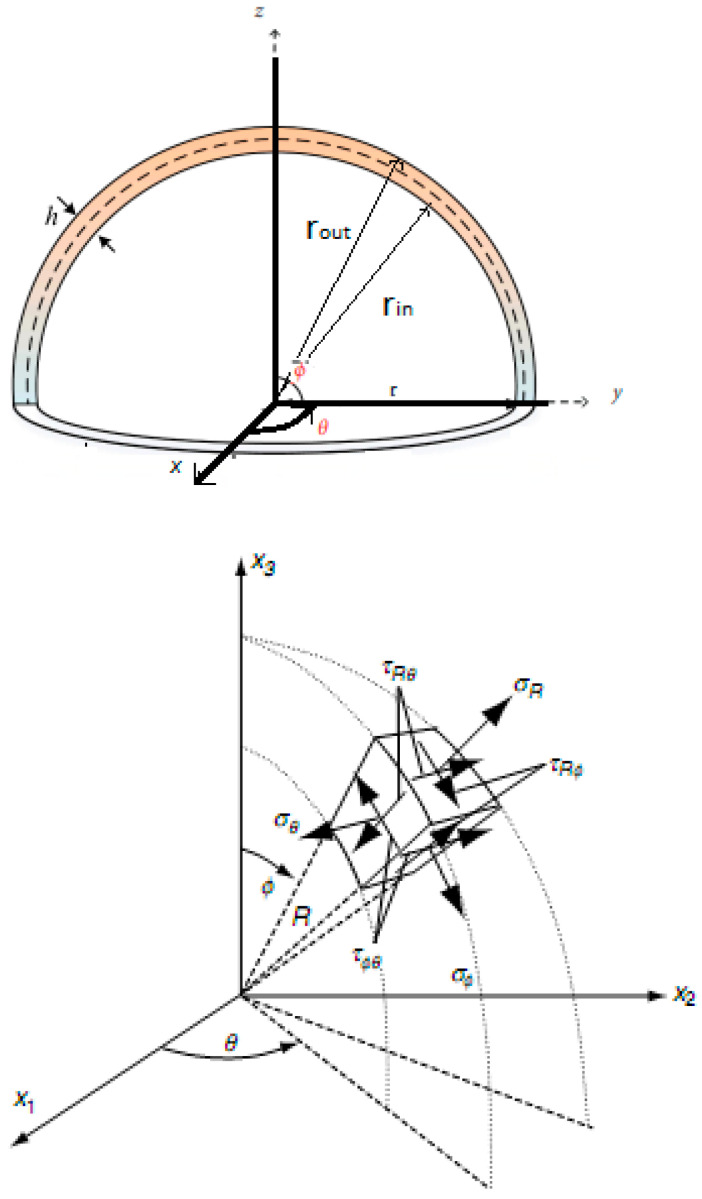
Main geometric parameters and assumptions for the spherical cap.

Let assume a spherical cap with uniform thickness h and mean radius of R. The outer $r_{out}=b$ and inner $r_{in}=a$ radii of the spherical cap are denoted as reported in Figure 1. The spherical cap is defined using the spherical coordinates that can be defined as follows: r, 0≤θ≤2π and 0≤ϕ≤π.

**Figure 2:**
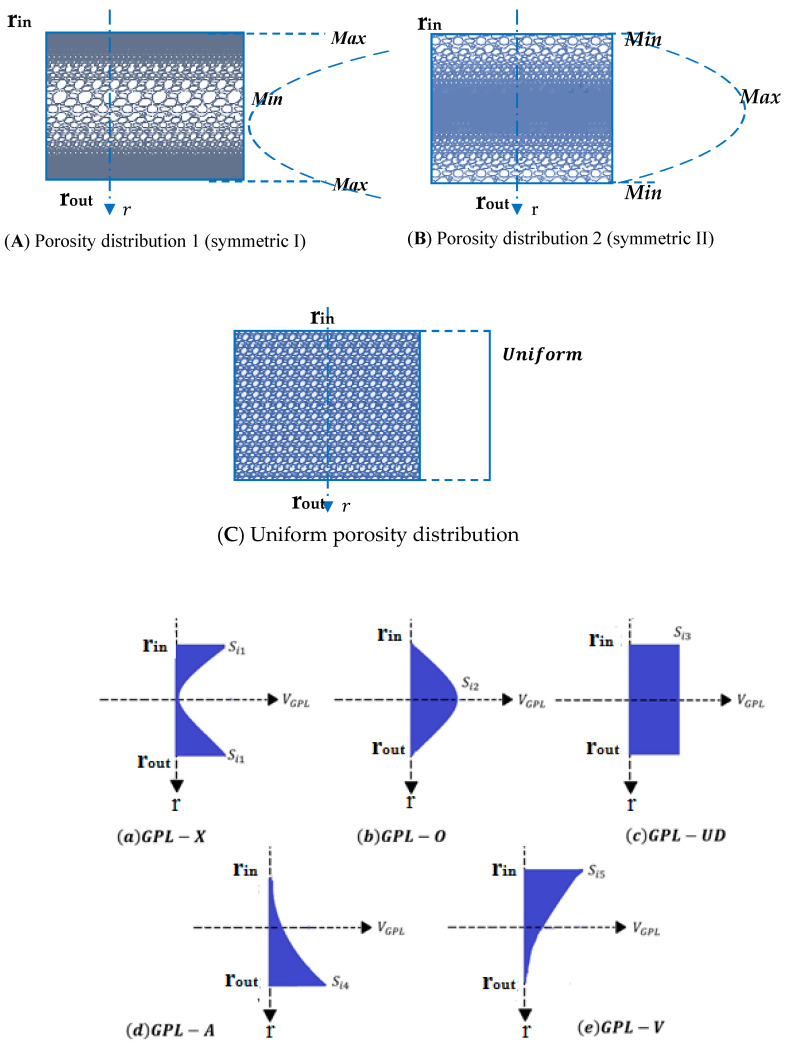
GPL distributions: (**A**) symmetric distribution I, (**B**) symmetric distribution II and (**C**) uniform distribution and patterns of porosities: (**a**) GPL-X, (**b**) GPL-O, (**c**) GPL-UD, (**d**) GPL-A, (**e**) GPL-V.

As can be seen in Figure 2, two types of non-uniform symmetric distributions and a uniform one are assumed in the present work, such that three different porosity profiles are here considered throughout the thickness of the spherical cap. In distribution 1, the porosity is nonlinear and symmetric. Furthermore, the distribution around the mid-radius is larger than the corresponding one around the external surfaces of the structure. In distribution 2, the porosity is also nonlinear and symmetric, but the porosity near the inner and outer surfaces of the spherical cap is higher than that one around the mid-radius. Equations (1)–(3) define mathematically the distributions of the material properties considering the effect of porosity, for the three selected distributions. At the same time, Figure 2 reports the five GPL distribution profiles throughout the spherical cap, thickness-wise, which are defined next [25,26]. More specifically, the mechanical properties refer to the mass density ρ(r), Young’s modulus E(r) and shear modulus G(r) of porous nanocomposite spherical caps [54,55,56,57,58].

- Porosity distribution 1
  (1) $$\left\{\begin{array}{c} E\left(r\right)=E^{*}\left[1-e_{0}\cos\left(\pi (\frac{r-r_{in}}{h}-\frac{1}{2})\right)\right]\\ G\left(r\right)=G^{*}\left[1-e_{0}\cos\left(\pi (\frac{r-r_{in}}{h}-\frac{1}{2})\right)\right]\\ \rho \left(r\right)=\rho^{*}\left[1-e_{m}\cos\left(\pi (\frac{r-r_{in}}{h}-\frac{1}{2})\right)\right] \end{array}\right.$$

- Porosity distribution 2
  (2) $$\left\{\begin{array}{c} E\left(r\right)=E^{*}\left[\left(1-e_{0}^{*}\left(1-\cos\left(\pi (\frac{r-r_{\mathrm{in}}}{h}-\frac{1}{2})\right)\right)\right]\right.\\ G\left(r\right)=G^{*}\left[\left(1-e_{0}^{*}\left(1-\cos\left(\pi (\frac{r-r_{\mathrm{in}}}{h}-\frac{1}{2})\right)\right)\right]\right.\\ \rho \left(r\right)=\rho^{*}\left[\left(1-e_{m}^{*}\left(1-\cos\left(\pi (\frac{r-r_{\mathrm{in}}}{h}-\frac{1}{2})\right)\right)\right]\right. \end{array}\right.$$

- Uniform porosity distribution

(3) $$\left\{\begin{array}{c} E\left(\varsigma \right)=E^{*}\alpha \\ G\left(\varsigma \right)=G^{*}\alpha \\ \rho \left(\varsigma \right)=\rho^{*}\alpha \end{array}\right.$$

where $\rho^{*}$, $E^{*}$ and $G^{*}$ refer to the mass density, Young’s modulus and shear modulus of the GPL spherical cap without interior cavities, respectively.

In addition, $e_{0}$ and $e_{0}^{*}\left(0\leq e_{0}\left(e_{0}^{*}\right)<1\right)$ refer to the coefficients of porosity for the first two profiles, respectively; $e_{m}$ and $e_{m}^{*}$ stand for the mass density coefficients for these two distributions, respectively; *α* and *α*′ are two parameters referring to a uniform porosity profile. For an increased size and density of the internal cavities, the porosity increases, with a subsequent reduction of the mechanical properties.

The relation between the elasticity modulus and density for an open-cell metal foam is assumed as [58,59]

(4) $$\frac{E\left(r\right)}{E^{*}}={\left(\frac{\rho \left(r\right)}{\rho^{*}}\right)}^{2}$$

which is adopted to derive the relation between porosity and mass density coefficients for various porosity patterns; i.e.,

(5) $$\{\begin{array}{ccc} 1-e_{m}\cos\left(\pi r\right) & = & \sqrt{1-e_{0}\cos\left(\pi r\right)}\\ 1-e_{m}^{*}\left(1-\cos\left(\pi r\right)\right) & = & \sqrt{1-e_{0}^{*}\left(1-\cos\left(\pi r\right)\right)}\\ \alpha^{\prime } & = & \sqrt{\alpha } \end{array}$$

Here, we assume that the masses of spherical caps with various porosity patterns and GPL dispersions are identical. To compare the stiffness of different distributions, indeed, the analyses should be implemented for shells with equal masses. Hence, the values of $e_{0}^{*}$ and α can be evaluated for a fixed value of $e_{0}$ [38,39], as

(6) $$\overset{r_{out}}{\underset{r_{in}}{\int }}\sqrt{1-e_{0}\cos\left(\pi r\right)}\;dr=\overset{r_{out}}{\underset{r_{in}}{\int }}\sqrt{1-e_{0}^{*}\left(1-\cos\left(\pi r\right)\right)}dr=\overset{h/2}{\underset{0}{\int }}\sqrt{\alpha }dr$$

According to Equation (6), the values of $e_{0}^{*}$ and α can be estimated for a fixed value of $e_{0}$, as shown in Table 1.

It can be seen that $e_{0}^{*}$ increases as the value of $e_{0}$ increases. When $e_{0}$ equals 0.6, $e_{0}^{*}$ becomes equal to 0.9612, which is near to the upper bound. Hereafter, $e_{0}\in \left[0,\;0.6\right]$ is used within the numerical investigation. According to the Halpin–Tsai micromechanics model [60], the elasticity modulus of nanocomposites without internal cavities is defined as

(7) $$E^{*}=\frac{3}{8}\left(\frac{1+\varepsilon_{L}^{GPL}\eta_{L}^{GPL}V_{GPL}}{1-\eta_{L}^{GPL}V_{GPL}}\right)E_{m}+\frac{5}{8}\left(\frac{1+\varepsilon_{W}^{GPL}\eta_{W}^{GPL}V_{GPL}}{1-\eta_{W}^{GPL}V_{GPL}}\right)$$

with

(8) $$\varepsilon_{L}^{GPL}=\frac{2l_{GPL}}{t_{GPL}}$$

(9) $$\varepsilon_{W}^{GPL}=\frac{2w_{GPL}}{t_{GPL}}$$

(10) $$\eta_{L}^{GPL}=\frac{E_{GPL}-E_{m}}{E_{GPL}+\varepsilon_{L}^{GPL}E_{m}}$$

(11) $$\eta_{W}^{GPL}=\frac{E_{GPL}-E_{m}}{E_{GPL}+\varepsilon_{W}^{GPL}E_{m}}$$

where indices m and GPL stand for properties of the metallic matrix and graphene platelets, respectively; $V_{GPL}$ is the volumetric content of GPLs; and $l_{GPL}$, $w_{GPL}$ and $t_{GPL}$ refer to the length, width and thickness of the nano-filler platelets, respectively.

Based on the rule of mixtures, the mass density and Poisson’s ratio of nanocomposite materials are defined as [61,62,63]

(12) $$\rho^{*}=\rho_{GPL}V_{GPL}+\rho_{m}\left(1-V_{GPL}\right)$$

(13) $$v^{*}=v_{GPL}V_{GPL}+v_{m}\left(1-V_{GPL}\right)$$

whereas

(14) $$G^{*}=\frac{E^{*}}{2\left(1+v^{*}\right)}$$

refers to the associated shear modulus. The volumetric content of GPLs, $V_{GPL}$*,* is assumed to vary throughout the spherical cap’s thickness, having five different dispersion patterns (see Figure 2):

(15) $$V_{GPL}(r)=\left\{\begin{array}{ll} S_{i1}\left(1-\cos\left(\pi \left(\frac{r-r_{in}}{h}-\frac{1}{2}\right)\right)\right) & GPL-X\\ S_{i2}\cos\left(\left(\pi \frac{r-r_{in}}{h}-\frac{1}{2}\right)\right) & GPL-O\\ S_{i3} & GPL-UD\\ S_{i4}\left(1-\cos\left(\pi \left(\frac{r-r_{in}}{2h}\right)\right)\right) & GPL-A\\ S_{i5}\cos\left(\pi \left(\frac{r-r_{in}}{2h}\right)\right) & GPL-V \end{array}\right\}$$

where $S_{i1},\;S_{i2},\;S_{i3},\;S_{i4}$ and $S_{i5}$ denote the upper limits of $V_{GPL}$; and subscript *i =* 1, 2 or 3 refers to various porosity distributions within each pattern. Moreover, $V_{GPL}^{T}$ stands for the total volumetric content of GPLs, which is defined in terms of the nanofiller weight fraction $\Delta_{GPL}$ in the following form:

(16) $$V_{GPL}^{T}=\frac{\Delta_{GPL}\rho_{m}}{\Delta_{GPL}\rho_{m}+\rho_{GPL}-\Delta_{GPL}\rho_{GPL}}$$

This is, in turn, used to derive $S_{i1},\;S_{i2},\;S_{i3},\;S_{i4}$ and $S_{i5}$ as

(17) $$V_{GPL}^{T}\int_{r_{in}}^{r_{out}}\frac{\rho (r)}{\rho^{*}}dr=\left\{ \begin{array}{l} S_{i1}\int_{r_{in}}^{r_{out}}[1-\cos\left(\pi (\frac{r-r_{in}}{h}-\frac{1}{2})\right)]\frac{\rho (r)}{\rho^{*}}dr\\ S_{i2}\int_{r_{in}}^{r_{out}}\cos\left(\pi (\frac{r-r_{in}}{h}-\frac{1}{2})\right)\frac{\rho (r)}{\rho^{*}}dr\\ S_{i3}\int_{r_{in}}^{r_{out}}\frac{\rho (r)}{\rho^{*}}dr\\ S_{i4}\int_{r_{in}}^{r_{out}}[1-\cos(\pi (\frac{r-r_{in}}{2h})]\frac{\rho (r)}{\rho^{*}}dr\\ S_{i5}\int_{r_{in}}^{r_{out}}\cos(\pi (\frac{r-r_{in}}{2h})\frac{\rho (r)}{\rho^{*}}dr \end{array}\right.$$

### 2.2. Governing Equations of the Problem

The stress–strain relations are defined in matrix form as

(18) σ=Dε

where the stress and strain field, together with the elasticity matrix D, read as follows:

(19) $$\sigma ={\left\{\begin{array}{cccccc} \sigma_{r} & \sigma_{\phi } & \sigma_{\theta } & \sigma_{r\phi } & \sigma_{\theta \phi } & \sigma_{r\theta } \end{array}\right\}}^{T}$$

(20) $$\varepsilon ={\left\{\begin{array}{cccccc} \varepsilon_{r} & \varepsilon_{\phi } & \varepsilon_{\theta } & \gamma_{r\phi } & \gamma_{\phi \theta } & \gamma_{r\theta } \end{array}\right\}}^{T}$$

(21) $$D=\frac{E^{*}(r)}{\left(1+\upsilon^{*})(1-2\upsilon^{*}\right)}\left[\begin{array}{cccccc} 1-\upsilon^{*} & \upsilon^{*} & \upsilon^{*} & 0 & 0 & 0\\ \upsilon^{*} & 1-\upsilon^{*} & \upsilon^{*} & 0 & 0 & 0\\ \upsilon & \upsilon & 1-\upsilon^{*} & 0 & 0 & 0\\ 0 & 0 & 0 & \frac{1-2\upsilon^{*}}{2} & 0 & 0\\ 0 & 0 & 0 & 0 & \frac{1-2\upsilon^{*}}{2} & 0\\ 0 & 0 & 0 & 0 & 0 & \frac{1-2\upsilon^{*}}{2} \end{array}\right]$$

where $\upsilon^{*}$ is the Poisson’s ratio and $E^{*}$ denotes the Young’s modulus that depends on the *r* coordinate. Based on the linear elasticity theory, the strain field in spherical coordinate is defined as

(22) $$\varepsilon =\varepsilon_{L}+\varepsilon_{NL}$$

with

(23) $$\varepsilon_{L}=\left[\begin{array}{c} \frac{\partial u}{\partial r}\\ \frac{1}{r}\left(u+\frac{\partial v}{\partial \phi }\right)\\ \frac{1}{r\sin\phi }\left(\frac{\partial w}{\partial \theta }+\sin\phi u+\cos\phi v\right)\\ \left(\frac{1}{r}\frac{\partial u}{\partial \phi }+\frac{\partial v}{\partial r}-\frac{v}{r}\right)\\ \frac{1}{r}\left(\frac{1}{\sin\phi }\frac{\partial v}{\partial \theta }+\frac{\partial w}{\partial \phi }-\cot\phi w\right)\\ \left(\frac{1}{r\sin\phi }\frac{\partial u}{\partial \theta }+\frac{\partial w}{\partial r}-\frac{w}{r}\right) \end{array}\right]$$

and

(24) $$\varepsilon_{NL}=\left[\begin{array}{c} \frac{1}{2}\left({\left(\frac{\partial u}{\partial r}\right)}^{2}+{\left(\frac{\partial v}{\partial r}\right)}^{2}+{\left(\frac{\partial w}{\partial r}\right)}^{2}\right)\\ \frac{1}{2}\left({\left(\frac{1}{r}\frac{\partial u}{\partial \phi }-v\right)}^{2}+{\left(\frac{1}{r}\left(u+\frac{\partial v}{\partial \phi }\right)\right)}^{2}+{\left(\frac{1}{r}\frac{\partial w}{\partial \phi }\right)}^{2}\right)\\ \frac{1}{2}\left({\left(\frac{1}{r\sin\phi }\left(\frac{\partial u}{\partial \theta }-w\sin\phi \right)\right)}^{2}+{\left(\frac{1}{r\sin\phi }\left(\frac{\partial v}{\partial \theta }-w\cos\phi \right)\right)}^{2}+{\left(\frac{1}{r\sin\phi }\frac{\partial w}{\partial \theta }+\frac{u}{r}+\frac{v\cot\phi }{r}\right)}^{2}\right)\\ \frac{1}{r}\frac{\partial u}{\partial r}\left(\frac{\partial u}{\partial \phi }-v\right)+\frac{1}{r}\frac{\partial v}{\partial r}\left(\frac{\partial v}{\partial \phi }+u\right)+\frac{1}{r}\frac{\partial w}{\partial r}\frac{\partial w}{\partial \phi }\\ \frac{1}{r^{2}\sin\phi }\left(\frac{\partial u}{\partial \phi }-v\right)\left(\frac{\partial u}{\partial \theta }-w\sin\phi \right)+\frac{1}{r^{2}\sin\phi }\left(\frac{\partial v}{\partial \phi }+u\right)\left(\frac{\partial v}{\partial \theta }-w\cos\phi \right)+\frac{1}{r}\frac{\partial w}{\partial \phi }\left(\frac{1}{r\sin\phi }\frac{\partial w}{\partial \theta }+\frac{u}{r}+\frac{v\cot\phi }{r}\right)\\ \frac{1}{r\sin\phi }\frac{\partial u}{\partial r}\left(\frac{\partial u}{\partial \theta }-w\sin\phi \right)+\frac{1}{r\sin\phi }\frac{\partial v}{\partial r}\left(\frac{\partial v}{\partial \theta }-w\cos\phi \right)+\frac{\partial w}{\partial r}\left(\frac{1}{r\sin\phi }\frac{\partial w}{\partial \theta }+\frac{u}{r}+\frac{v\cot\phi }{r}\right) \end{array}\right]$$

In addition, *u*, *v* and *w* define the kinematic components along *r*, ϕ and θ directions, respectively. According to the above-mentioned relations, the linear strain relation can be rewritten as

(25) $$\varepsilon_{L}=ℒQ$$

where **Q** is the displacements vector and ℒ is an operator matrix involving the partial derivatives of a function

(26) $$Q={\left\{\begin{array}{ccc} u & v & w \end{array}\right\}}^{T}$$

(27) $$ℒ={\left[\begin{array}{cccccc} \partial_{r} & 1/r & 1/r & 1/2r\partial_{\phi } & 0 & \frac{1}{2r\sin\phi }\partial_{\theta }\\ 0 & 1/r\partial_{\phi } & 1/r\cot\phi & \frac{1}{2}\partial_{r}-\frac{1}{2r} & \frac{1}{2r\sin\phi }\partial_{\theta } & 0\\ 0 & 0 & \frac{1}{r\sin\phi }\partial_{\theta } & 0 & \partial_{\phi }-\cot\phi & \partial_{r}-\frac{1}{r} \end{array}\right]}^{T}$$

## 3. Finite Element Modeling

A FEM-based approach is now adopted to solve the governing equations of the problem, where the spherical cap is divided into 8–node linear brick elements. For element (e), the 3D kinematic field is approximated as

(28) $$Q^{\left(e\right)}=\Phi \Lambda^{\left(e\right)}$$

where Φ is the matrix of linear shape functions in spherical coordinates, whereas $\Lambda^{\left(e\right)}$ refers to the nodal displacement vector of the element, which is defined as

(29) $$\Phi =\left[\begin{array}{ccccccc} \Phi_{1} & 0 & 0 & \cdots & \Phi_{8} & 0 & 0\\ 0 & \Phi_{1} & 0 & \cdots & 0 & \Phi_{8} & 0\\ 0 & 0 & \Phi_{1} & \cdots & 0 & 0 & \Phi_{8} \end{array}\right]$$

(30) $$\Lambda^{\left(e\right)}={\left\{\begin{array}{ccccccc} U_{1} & V_{1} & W_{1} & \cdots & U_{8} & V_{8} & W_{8} \end{array}\right\}}^{T}$$

The components of Φ are

(31) $$\Phi_{i}=\frac{1}{V}\Gamma X$$

V being the volume of each element; i.e.,

(32) $$V=\left|\begin{array}{cccccccc} 1 & \xi_{1} & \eta_{1} & \zeta_{1} & \xi_{1}\eta_{1} & \xi_{1}\zeta_{1} & \eta_{1}\zeta_{1} & \xi_{1}\eta_{1}\zeta_{1}\\ 1 & \xi_{2} & \eta_{2} & \zeta_{2} & \xi_{2}\eta_{2} & \xi_{2}\zeta_{2} & \eta_{2}\zeta_{2} & \xi_{2}\eta_{2}\zeta_{2}\\ 1 & \xi_{3} & \eta_{3} & \zeta_{3} & \xi_{3}\eta_{3} & \xi_{3}\zeta_{3} & \eta_{3}\zeta_{3} & \xi_{3}\eta_{3}\zeta_{3}\\ 1 & \xi_{4} & \eta_{4} & \zeta_{4} & \xi_{4}\eta_{4} & \xi_{4}\zeta_{4} & \eta_{4}\zeta_{4} & \xi_{4}\eta_{4}\zeta_{4}\\ 1 & \xi_{5} & \eta_{5} & \zeta_{5} & \xi_{5}\eta_{5} & \xi_{5}\zeta_{5} & \eta_{5}\zeta_{5} & \xi_{5}\eta_{5}\zeta_{5}\\ 1 & \xi_{6} & \eta_{6} & \zeta_{6} & \xi_{6}\eta_{6} & \xi_{6}\zeta_{6} & \eta_{6}\zeta_{6} & \xi_{6}\eta_{6}\zeta_{6}\\ 1 & \xi_{7} & \eta_{7} & \zeta_{7} & \xi_{7}\eta_{7} & \xi_{7}\zeta_{7} & \eta_{7}\zeta_{7} & \xi_{7}\eta_{7}\zeta_{7}\\ 1 & \xi_{8} & \eta_{8} & \zeta_{8} & \xi_{8}\eta_{8} & \xi_{8}\zeta_{8} & \eta_{8}\zeta_{8} & \xi_{8}\eta_{8}\zeta_{8} \end{array}\right|$$

and

(33) $$\Gamma_{ij}={\left(-1\right)}^{i+j}\left|A_{ij}\right|$$

(34) $$X={\left\{1,\xi ,\eta ,\zeta ,\xi \eta ,\xi \zeta ,\eta \zeta ,\xi \eta \zeta \right\}}^{T}$$

It is also

(35) $$\begin{array}{ccc} \xi =r\cos\theta \sin\phi , & \eta =r\sin\theta \sin\phi , & \zeta =r\cos\phi \end{array}$$

(36) $$\begin{array}{ccc} \xi_{i}=r_{i}\cos\theta_{i}\sin\phi_{i}, & \eta_{i}=r_{i}\sin\theta_{i}\sin\phi_{i}, & \zeta_{i}=r_{i}\cos\phi_{i} \end{array}$$

where $r_{i}$, $\theta_{i}$ and $\phi_{i}$ are the nodal coordinates and $A_{ij}$ is obtained by elimination of the *i*th row and *j*th column from *V*. Substituting Equation (26) into Equation (23) gives the strain matrix of element (e) as

(37) $$\varepsilon_{L}{}^{\left(e\right)}=B\Lambda^{\left(e\right)}$$

where

(38) $$B=ℒ\Phi^{\left(e\right)}$$

The FEM-based governing equations are determined from the principle of virtual work, where the potential energy *U* and virtual work of external loads δW are defined as

(39) δΠ=δU−δW=0

(40) $$\delta u=\int_{V^{\left(e\right)}}^{\;}{\left(\delta \varepsilon^{\left(e\right)}\right)}^{T}\sigma^{\left(e\right)}dV\;$$

(41) $$\delta W={\left(\int_{A}^{}{\bar{\sigma }}_{{}_{rr}}\delta u\;dA\right)}_{r=b}^{}$$

A being the area under the external radial load $\bar{\sigma_{rr}}=1$, which is subjected to the external surface of a spherical cap. In a pre-buckling state, the displacement field can be considered to be small, and the nonlinear terms of strain–displacement relations vanish. Therefore, one may write

(42) $$\delta U=\int_{V^{\left(e\right)}}\delta {\left(B\Lambda^{\left(e\right)}\right)}^{T}D(B\Lambda^{\left(e\right)})dV=\delta \Lambda^{{\left(e\right)}^{T}}K\Lambda^{\left(e\right)}$$

Therefore, based on the principle of virtual work, the static balance equation of the problem for each element in a pre-buckling state takes the following form

(43) $$\delta \Lambda^{{\left(e\right)}^{T}}\int_{V^{\left(e\right)}}B^{T}DBdV\Lambda^{\left(e\right)}=\delta \Lambda^{{\left(e\right)}^{T}}\int_{A^{\left(e\right)}}\varphi^{T}pdA$$

Equation (43) in compact form can be written as

(44) $$K^{\left(e\right)}\Lambda^{\left(e\right)}=F^{\left(e\right)}$$

where $K^{\left(e\right)}$ is the linear stiffness matrix and $F^{\left(e\right)}$ is the force matrix for each element, defined as

(45) $$K^{\left(e\right)}=\int_{V^{\left(e\right)}}B^{T}DB\;dV$$

(46) $$F^{\left(e\right)}=\int_{A^{\left(e\right)}}{}_{}\Phi^{T}pdA$$

(47) $$p={\left\{\begin{array}{c} \bar{\sigma_{rr}}\\ 0\\ 0 \end{array}\right\}}_{r=b}$$

By assembling each element matrix, the equilibrium equation of the spherical cap in the pre-buckling state is obtained as

(48) KΛ=F

whose solution is determined in terms of the strain field in pre-buckling state for $\bar{\sigma_{rr}}=1$. Afterward, the stress field due to these deformations is used in the geometric stiffness matrix, as detailed in the following. Finally, in order to determine the governing equations of an instability problem, the following equation can be used:

(49) $$\delta \left(\delta \Pi \right)=\delta^{2}\Pi =0$$

Therefore, based on Equations (44) and (48), it is

(50) $$\delta \Lambda^{\left(e\right)}{}^{T}K^{\left(e\right)}\delta \Lambda^{\left(e\right)}+\delta^{2}\Pi_{Ext.}=0$$

In line with Equation (22), the linear and nonlinear terms of the kinematic relations have been considered in the strain energy of the shell. In the pre-buckling state, the radial displacement components or large deformations can be assumed to be small, whereas only the linear strain terms appear. At the buckling state, instead, the nonlinear kinematic relations have to be considered. Therefore, the following relation can be obtained:

(51) $$\begin{array}{l} U=\Pi_{Ext.}=\frac{1}{2}\int_{V}{\left(\varepsilon^{nl}\right)}^{T}\sigma dV \end{array}$$

$\Pi_{Ext.}$ can be rewritten as

(52) $$\Pi_{Ext.}=\frac{1}{4}\int_{V}\psi^{T}\Theta \psi dV=\frac{1}{4}\int_{V}{\left(\Xi Q\right)}^{T}\Theta \left(\Xi Q\right)dV$$

where

$$\psi^{T}={\left(\begin{array}{ccccccccc} \frac{\partial u}{\partial r} & \frac{1}{r}\frac{\partial u}{\partial \phi }-v & \frac{1}{r\;\sin\phi }\left(\frac{\partial u}{\partial \theta }-w\sin\phi \right) & \frac{\partial v}{\partial r} & \frac{1}{r}\left(u+\frac{\partial v}{\partial \phi }\right) & \frac{1}{r\sin\phi }\left(\frac{\partial v}{\partial \theta }-w\cos\phi \right) & \frac{\partial w}{\partial r} & \frac{1}{r}\frac{\partial w}{\partial \phi } & \frac{1}{r\sin\phi }\frac{\partial w}{\partial \theta }+\frac{u}{r}+\frac{v\cot\phi }{r} \end{array}\right)}_{1*9}\;\psi_{9*1}=\Xi_{9*3}\;Q$$

and

$$\begin{array}{l} \Xi =\left[\begin{array}{ccc} \frac{\partial }{\partial r} & 0 & 0\\ \frac{1}{r}\frac{\partial }{\partial \phi } & -\frac{1}{r} & 0\\ \frac{1}{r\;\sin\phi }\frac{\partial }{\partial \theta } & 0 & -\frac{1}{r}\\ 0 & \frac{\partial }{\partial r} & 0\\ \frac{1}{r} & \frac{1}{r}\frac{\partial }{\partial \phi } & 0\\ 0 & \frac{1}{r\;\sin\phi }\frac{\partial }{\partial \theta } & -\frac{\cot\phi }{r}\\ 0 & 0 & \frac{\partial }{\partial r}\\ 0 & 0 & \frac{1}{r}\frac{\partial }{\partial \phi }\\ \frac{1}{r} & \frac{\cot\phi }{r} & \frac{1}{r\;\sin\phi }\frac{\partial }{\partial \theta } \end{array}\right] \end{array}$$

$$\begin{array}{l} \Theta =\left[\begin{array}{ccc} S^{0} & 0_{3\times 3} & 0_{3\times 3}\\ 0_{3\times 3} & S^{0} & 0_{3\times 3}\\ 0_{3\times 3} & 0_{3\times 3} & S^{0} \end{array}\right]\\ S^{0}=\left[\begin{array}{ccc} \sigma^{0}{}_{rr^{}} & \sigma^{0}{}_{r\phi } & \sigma^{0}{}_{r\theta }\\ \sigma^{0}{}_{r\phi } & \sigma^{0}{}_{\phi \phi } & \sigma^{0}{}_{\phi \theta }\\ \sigma^{0}{}_{r\theta } & \sigma^{0}{}_{\phi \theta } & \sigma^{0}{}_{\theta \theta } \end{array}\right] \end{array}$$

In the last relation, $S^{0}$ refers to the stresses obtained in pre-buckling state. By substituting $Q^{\left(e\right)}=\Phi \Lambda^{\left(e\right)}$, we obtain

(53) $$\psi =\Xi_{}\;\Phi \Lambda^{\left(e\right)}$$

where

(54) $$\Omega =\Xi_{}\;\Phi$$

More in detail, it is

(55) $$\begin{array}{l} \Omega =\left[\begin{array}{ccccccc} \frac{\partial \Phi_{1}}{\partial r} & 0 & 0 & \cdots & \frac{\partial \Phi_{8}}{\partial r} & 0 & 0\\ \frac{1}{r}\frac{\partial \Phi_{1}}{\partial \phi } & -\frac{\Phi_{1}}{r} & 0 & \cdots & \frac{1}{r}\frac{\partial \Phi_{8}}{\partial \phi } & -\frac{\Phi_{8}}{r} & 0\\ \frac{1}{r\;\sin\phi }\frac{\partial \Phi_{1}}{\partial \theta } & 0 & -\frac{\Phi_{1}}{r} & \cdots & \frac{1}{r\;\sin\phi }\frac{\partial \Phi_{8}}{\partial \theta } & 0 & -\frac{\Phi_{8}}{r}\\ 0 & \frac{\partial \Phi_{1}}{\partial r} & 0 & \cdots & 0 & \frac{\partial \Phi_{8}}{\partial r} & 0\\ \frac{\Phi_{1}}{r} & \frac{1}{r}\frac{\partial \Phi_{1}}{\partial \phi } & 0 & \cdots & \frac{\Phi_{8}}{r} & \frac{1}{r}\frac{\partial \Phi_{8}}{\partial \phi } & 0\\ 0 & \frac{1}{r\;\sin\phi }\frac{\partial \Phi_{1}}{\partial \theta } & -\frac{\Phi_{1}\;\cot\phi }{r} & \cdots & 0 & \frac{1}{r\;\sin\phi }\frac{\partial \Phi_{8}}{\partial \theta } & -\frac{\Phi_{8}\;\cot\phi }{r}\\ 0 & 0 & \frac{\partial \Phi_{1}}{\partial r} & \cdots & 0 & 0 & \frac{\partial \Phi_{8}}{\partial r}\\ 0 & 0 & \frac{1}{r}\frac{\partial \Phi_{1}}{\partial \phi } & \cdots & 0 & 0 & \frac{1}{r}\frac{\partial \Phi_{8}}{\partial \phi }\\ \frac{\Phi_{1}}{r} & \frac{\Phi_{1}\;\cot\phi }{r} & \frac{1}{r\;\sin\phi }\frac{\partial \Phi_{1}}{\partial \theta } & \cdots & \frac{\Phi_{8}}{r} & \frac{\Phi_{8}\;\cot\phi }{r} & \frac{1}{r\;\sin\phi }\frac{\partial \Phi_{8}}{\partial \theta } \end{array}\right] \end{array}$$

Thus, according to Equation (50), we have

(56) $$\delta^{2}\Pi =\delta \;(\Lambda^{(e)T})K^{\left(e\right)}\delta \;(\Lambda^{\left(e\right)})+\;\delta \;(\Lambda^{(e)T})(\int_{V^{\left(e\right)}}\Omega^{T}\Theta \Omega dV)\;\delta \;(\Lambda^{\left(e\right)})=0$$

Equation (50) can be redefined in the following form:

(57) $$\delta (\Lambda^{(e)T})(K^{\left(e\right)}+K_{G}^{\left(e\right)})\delta (\Lambda^{\left(e\right)})=0$$

After the assembly of the element matrices, the following determinant should be assumed as null for the structure.

(58) $$\left|K+K_{G}\right|=0$$

(59) $$\left|\int_{V^{\left(e\right)}}B^{T}DBdV+\lambda_{Cr}\int_{V}{}_{\left(e\right)}\Omega^{T}\Theta \Lambda \Omega dV\right|=0\;$$

Note that K and $K_{G}$ refer to the linear stiffness matrix and geometric stiffness matrix, respectively, which are computed using the Gauss 8-point numerical integration rules.

Hereafter, we assume the following clamped boundary conditions:

- For a spherical cap with *θ* = 180°, *ϕ* = 180°, *u*, *v*, *w* (*r*, *θ* and *ϕ* = 0, 180°), $\bar{\sigma_{\mathrm{rr}}}$ = 1 at *r* = *b*.
- For a spherical cap with *θ* = 180°, *ϕ* = 90°, *u*, *v*, *w* (*r*, *θ* and *ϕ* = 0, 90°), $\bar{\sigma_{\mathrm{rr}}}$ = 1 at *r* = *b*.

## 4. Numerical Results and Discussion

In this section, we discuss the numerical results in terms of buckling loads of an FG-GPL, porous spherical cap with clamped boundary conditions, for various volume or weight fractions of GPL, and for different GPL distribution patterns, porosity distributions and coefficients, along with two polar angles of the FG-GPL, porous spherical shell.

### 4.1. Validation

In order to verify our results, we started the analysis with a comparative evaluation of the buckling predictions using the commercial Ansys Workbench code, for an isotropic homogeneous spherical cap. Hence, the following changes were considered in our study: *e*_0_ = 0, *γ_GPL_* = 0. As far as the mechanical properties are concerned, we assumed *E_m_* = 130 GPa, *ρ_m_* = 8960 kg/m^3^, *ν_m_* = 0.34 for the copper material. As geometrical dimensions, we assumed: *a* = 0.225 m, *b* = 0.25 m, *θ* = 180°, *ϕ* = 180°, 90°. In this way, the FG-GPL porous structure changes to an isotropic homogenous structure. The comparison between our results and predictions from Ansys Workbench is shown in Table 2, with an excellent agreement among them.

### 4.2. Parametric Analysis of the Buckling Load

We now study the effects of two polar angles, porosity coefficient, porosity distribution, GPL patterns and the weight fraction of GPL nanofillers (for the first time) on the buckling load of an FG-GPL, porous spherical cap with clamped boundaries. Hence, the following geometrical properties are considered: *a* = 0.225 m, *b* = 0.25 m, *θ* = 180°, *ϕ* = 180°, 90°. The materials is characterized by the following mechanical properties: *E_m_* = 130 GPa, *ρ_m_* = 8960 kg/m^3^ and *ν_m_* = 0.34 for the copper material [28]; and *E_GPL_* = 1.01 TPa, *ρ_GPL_* = 1062.5 kg/m^3^, *ν_GPL_* = 0.186, *w_GPL_* = 1.5 μm, *l_GPL_* = 2.5 μm and *t_GPL_* = 1.5 nm for GPLs.

Table 3 indicates the influences of two types of polar angles and various GPL patterns on the buckling load of the FG-GPL-reinforced, porous spherical structure, under the assumptions PD3, *e*_0_ = 0.2 and *γ* = 0.01 wt%. The extreme values of buckling load are related to GPLX and GPL-O distributions, respectively, which means that, when GPLs accumulate around the inner and outer surfaces of the shell, the stiffness reaches its highest value. Moreover, when GPLs are sparser around the outer and inner surfaces of the shell, the minimum buckling load is obtained. Note also that the critical buckling loads for a GPLA and GPL-V distributions are approximately the same. The results also show that the surface area of the shell increases by increasing the polar angle, and there are consecutive increases in the structural stiffness and buckling load.

The influences of two polar angles and various porosity distributions are reported in Table 4 (GPLX, *e*_0_ = 0.4, *γ* = 0.01 wt%), which shows that the maximum and minimum buckling loads belong to PD1 and PD2 distributions, respectively. PD1 provides higher structural stiffness, and PD2 gives the minimum stiffness of the spherical cap shell. The main difference between the extreme values of critical buckling load is approximately 90% for different porosity patterns. This means that the porosity distribution has a considerable effect on the buckling loads of FG-GPL, porous, spherical cap shells.

The influences of two polar angles and weight fractions of nanofillers on the buckling loads of FG-GPL, porous spherical shell (PD1, *e*_0_ = 0.5, GPLX) are given in Table 5. Note that, by increasing the weight fraction of GPLs, the critical buckling loads of shell increases significantly (approximately 100%), along with a small variation of the structural mass. This issue can be useful for aerospace structures where the high stiffness and low density are extremely important.

**Figure 3:**
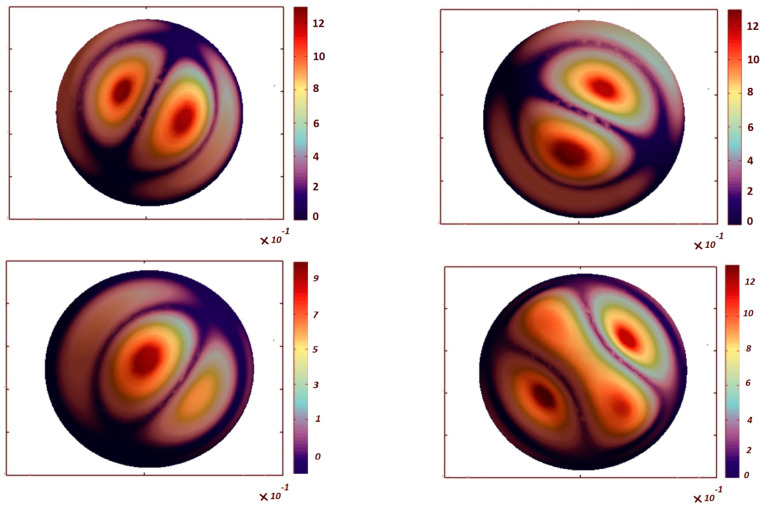

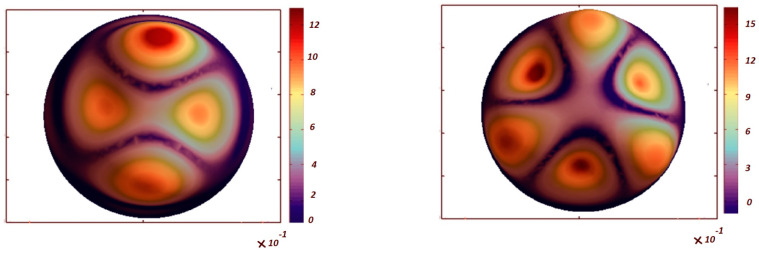
The first six buckling mode shapes of an FG-GPL, porous spherical cap (ϑ = 180°, ϕ = 180°, GPL-X, PD1, *e*_0_ = 0.4, *γ* = 0.01 wt%).

**Figure 4:**
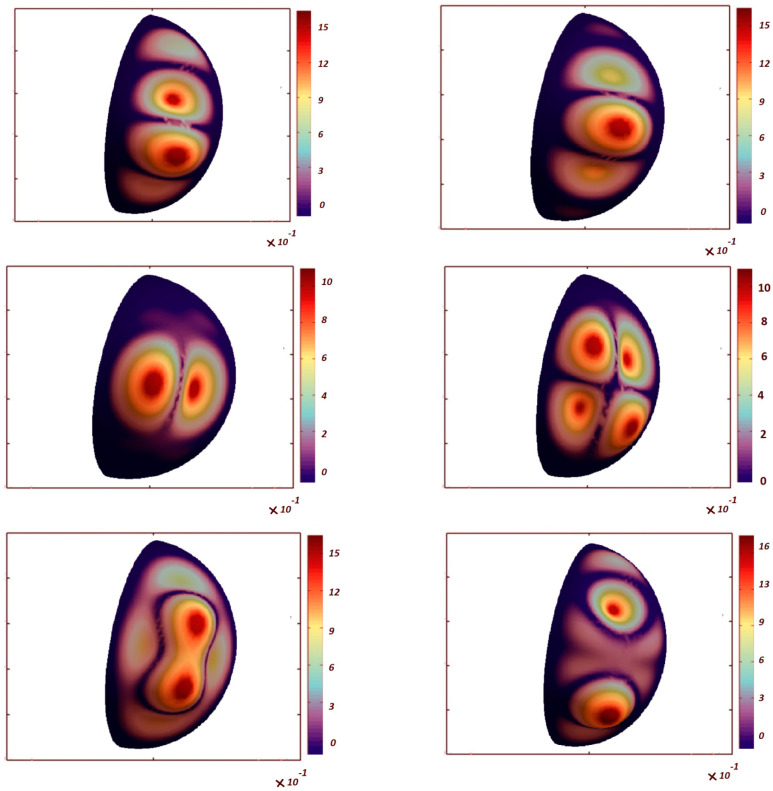
The first six buckling mode shapes of FG-GPL, porous spherical cap (ϑ = 180°, *ϕ* = 90°, GPL-X, PD1, *e*_0_ = 0.4, *γ* = 0.01 wt%).

Table 6 shows the effect of the porosity coefficient on the critical buckling loads of FG-GPL, porous spherical shells (PD1, *γ* = 0.01 wt%, GPLX). When the porosity of the structure increases, the critical buckling load of FG–GPL porous spherical shells decreases, because of the decreased structural stiffness. The comparative evaluation of Table 3, Table 4, Table 5 and Table 6 denotes that the influence of the porosity coefficient on the critical buckling load is lower than the GPL pattern and weight fraction of the nanofiller (its impact is approximately equal to 12.5%). On the other hand, the effects of the GPL pattern, porosity distribution and weight fraction of nanofillers on the critical buckling loads of FG–GPL, porous spherical shells are more pronounced than the porosity coefficient. The first six buckling mode shapes are shown in Figure 3 and Figure 4 for the clamped spherical caps with ϑ = 180°, ϕ = 180° and 90°, respectively. The figures clearly show that the first two buckling mode shapes and loads for each polar angle of spherical cap are the same. It is also observable that the number of buckling waves increases for higher buckling modes.

## 5. Concluding Remarks

The present work has studied the buckling responses of spherical caps made of FG porous materials reinforced by GPLs. Three different porosity distributions and five GPL patterns have been considered, along with the shell thickness. The equilibrium equations for the pre-buckling state have been determined according to the linear 3D elasticity theory and the principle of virtual work, whereas the buckling load associated with the problem has been computed according to the nonlinear Green strain field and generalized geometric stiffness concepts. We have studied the influence of the GPL pattern, weight fraction of nanofillers, porosity coefficient, porosity distribution and polar angles on the buckling loads of porous spherical cap made of FG-GPLs. Based on a large systematic investigation, the final remarks can be summarized as follows:

(a) The maximum and minimum buckling loads seem to be reached for GPL-X and GPL-O distributions, respectively.
(b) The maximum and minimum buckling loads belong to the PD1 and PD2 cases, respectively.
(c) The difference between the maximum and minimum critical buckling loads for different porosity distributions is approximately equal to 90%, and the buckling loads of the selected structure increase considerably (approximately of 100%) with an increase in the weight fraction of GPLs.
(d) The effect of the porosity coefficient on the critical buckling load for porous spherical cap shells made of FG-GPLs is lower than the weight fraction of the nanofillers, being approximately equal to 12.5%.

Such results could be useful for designing similar shell members with optimized mechanical properties and structural performances, as required by various engineering applications.

## Figures and Tables

**Table 1:** Porosity coefficients for different distributions.

$e_{0}$	$e_{0}^{*}$	α
0.1	0.1738	0.9361
0.2	0.3442	0.8716
0.3	0.5103	0.8064
0.4	0.6708	0.7404
0.5	0.8231	0.6733
0.6	0.9612	0.6047

**Table 2:** Comparison of buckling loads between present study and Ansys Workbench.

Polar Angle		$\omega_{1}$	$\omega_{2}$	$\omega_{3}$	$\omega_{4}$	$\omega_{5}$	$\omega_{6}$
	(Ansys Workbench)	2.890	2.901	2.990	3.001	3.012	3.078
180°	(Present)	2.908	2.924	3.008	3.021	3.033	3.132
	(Ansys Workbench)	1.861	1.867	2.110	2.101	2.159	2.299
90°	(Present)	1.873	1.875	2.180	2.211	2.222	2.320

**Table 3:** Buckling loads (GPa) of FG-GPL, porous spherical caps for various polar angles and GPL patterns (PD3, *e*_0_ = 0.2, *γ* = 0.01 wt%).

GPL Pattern	ϕ	*λ*_1_	*λ*_2_	*λ*_3_	*λ*_4_	*λ*_5_	*λ*_6_
	90°	3.174	3.174	3.670	3.775	3.778	3.939
GPL-X	180°	4.450	4.462	4.572	4.600	4.617	4.798
	90°	1.898	1.901	2.227	2.254	2.264	2.354
GPL-A	180°	2.914	2.925	2.997	3.009	3.018	3.109
	90°	1.885	1.888	2.212	2.238	2.248	2.336
GPL-V	180°	2.906	2.917	2.988	3.001	3.009	3.098
	90°	1.653	1.657	1.949	1.950	1.958	2.018
GPL-O	180°	2.731	2.747	2.801	2.821	2.824	2.894
	90°	1.903	1.906	2.233	2.259	2.270	2.359
GPL-UD	180°	2.926	2.937	3.009	3.022	3.030	3.121

**Table 4:** Buckling loads (GPa) of FG-GPL, porous spherical caps for various polar angles and porosity distributions (GPLX, *e*_0_ = 0.4, *γ* = 0.01wt%).

Porosity Distribution	ϕ	*λ*_1_	*λ*_2_	*λ*_3_	*λ*_4_	*λ*_5_	*λ*_6_
	90°	2.842	2.842	3.268	3.346	3.359	3.473
PD1	180°	3.964	3.974	4.076	4.092	4.111	4.259
	90°	1.581	1.584	1.862	1.863	1.870	1.926
PD2	180°	2.613	2.632	2.684	2.703	2.707	2.774
	90°	2.249	2.249	2.607	2.677	2.683	2.799
PD3	180°	3.191	3.199	3.280	3.297	3.310	3.434

**Table 5:** Buckling loads (GPa) of an FG-GPL, porous spherical cap for various polar angle and weight fractions of GPL nano-fillers (PD1, *e*_0_ = 0.5, GPLX).

Weight Fraction of Nano-Fillers (%wt)	ϕ	*λ*_1_	*λ*_2_	*λ*_3_	*λ*_4_	*λ*_5_	*λ*_6_
	90°	1.458	1.459	1.696	1.737	1.744	1.819
0%	180°	2.102	2.107	2.162	2.171	2.180	2.258
	90°	2.191	2.191	2.528	2.601	2.602	2.710
0.5%	180°	3.048	3.056	3.130	3.151	3.163	3.290
	90°	2.744	2.746	3.187	3.261	3.268	3.403
1%	180°	3.927	3.940	4.032	4.062	4.076	4.246

**Table 6:** Buckling loads (GPa) of FG-GPL, porous spherical caps for various polar angles and porosity coefficients (PD1, *γ* = 0.01 wt%, GPLX).

$e_{0}$	ϕ	*λ*_1_	*λ*_2_	*λ*_3_	*λ*_4_	*λ*_5_	*λ*_6_
	90°	3.262	3.262	3.780	3.881	3.889	4.055
0.2	180°	4.626	4.635	4.752	4.777	4.794	4.975
	90°	2.842	2.842	3.268	3.346	3.359	3.473
0.4	180°	3.964	3.974	4.076	4.092	4.111	4.259
	90°	2.744	2.746	3.187	3.261	3.268	3.403
0.5	180°	3.927	3.940	4.032	4.062	4.076	4.246

## Data Availability

No new data were created or analyzed in this study. Data sharing is not applicable to this article.
